# Complete genome sequence of *Edwardsiella tarda* strain GBS0709 isolated from a Japanese patient with the acute motor axonal neuropathy subtype of Guillain–Barré syndrome

**DOI:** 10.1128/mra.00456-24

**Published:** 2024-07-31

**Authors:** Masayuki Imajoh, Masahiro Mori, Takeshi Shimizu, Yume Koizumi, Yuma Kobayashi, Miyu Kawahara, Masanori Daibata

**Affiliations:** 1 Department of Marine Resource Science, Faculty of Agriculture and Marine Science, Kochi University, Kochi, Japan; 2 Department of Neurology, Graduate School of Medicine, Chiba University, Chiba, Japan; 3 Department of Molecular Infectiology, Graduate School of Medicine, Chiba University, Chiba, Japan; 4 Department of Microbiology and Infection, Kochi Medical School, Kochi University, Kochi, Japan; University of Maryland School of Medicine, Baltimore, Maryland, USA

**Keywords:** Guillain-Barré syndrome, acute motor axonal neuropathy, *Edwardsiella tarda*

## Abstract

The complete genome sequence of *Edwardsiella tarda* strain GBS0709, isolated from an 81-year-old Japanese patient with the acute motor axonal neuropathy subtype of Guillain–Barré syndrome, was determined. It comprised a 3,632,068 bp circular chromosome and a 5,386 bp plasmid. The overall guanine and cytosine content was 57.3%.

## ANNOUNCEMENT

Guillain–Barré syndrome (GBS) is a severe acute immune-mediated paralytic neuropathy affecting humans worldwide (1). In 2019, 150,095 cases of GBS were reported globally, with an age-standardized point prevalence of 0.8–6.4 per 100,000 population of which Japan exhibited the highest prevalence (2). The primary infectious agents associated with GBS include *Campylobacter jejuni*, *Mycoplasma pneumoniae*, and cytomegalovirus (3). Notably, cases of acute motor axonal neuropathy (AMAN), a subtype of GBS, are often reported in Japan, with *C*. *jejuni* infections exclusively eliciting the subtype (4, 5). Strain GBS0709 was isolated on bromothymol blue agar (Eiken Chemical, Japan) by overnight incubation under aerobic conditions at 35℃ from the feces of an 81-year-old Japanese patient with AMAN and identified as *Edwardsiella tarda* by matrix-assisted laser desorption/ionization time-of-flight mass spectrometry (6). Here, we report the complete genome sequence of GBS0709.

Frozen stock of GBS0709 was cultured on brain heart infusion (BHI) agar (BD Difco, USA) overnight at 37°C. A single colony was selected, inoculated into 10 mL of BHI broth, and incubated with shaking overnight at 37°C. Following centrifugation at 16,000 × *g* for 2 min, genomic DNA was extracted from bacterial pellets using the Wizard HMW DNA Extraction Kit (Promega, USA) following the manufacturer’s instructions and used for Illumina and Nanopore sequencing. Regarding Illumina sequencing, genomic DNA was enzymatically fragmented and prepared to achieve an average size of 330 bp using the QIAseq FX DNA Library Kit (Qiagen, USA) following the manufacturer’s instructions. After adaptor ligation, 20 pM pooled libraries were sequenced on an Illumina MiSeq (MiSeq Reagent Kit v3; 150 cycles, USA), generating 75 bp paired-end reads. Miseq raw reads with Phred-encoded quality scores above 30 were selected for assembly using Trim Galore version 0.6.5 (7). Regarding Nanopore sequencing, genomic DNA was treated with the 1D Ligation Sequencing Kit (SQK-LSK109, Oxford Nanopore Technologies, USA) with Native Barcoding Expansion (EXP-NBD104) without shearing and size selection to avoid fragmentation. After adaptor ligation, the pooled libraries were sequenced on a MinION with an R9.4.1 flow cell using MinKNOW software version 22.05.5. Base calling was performed using Guppy version 4.2.3 in the accurate mode implemented in MinION software. MinION raw reads below a minimum quality score of 8 were discarded using NanoFilt version 2.8.0 (8). Unicycler version 0.4.8 (9) was used for the hybrid assembly of Miseq and MinION reads. Genome error correction, circularization, and rotation were implemented in the Unicycler pipeline. Gene annotation, taxonomy validation, and genomic average nucleotide identity (gANI) calculation were performed using the DDBJ Fast Annotation and Submission Tool version 1.2.0 (10). Default parameters were used for all software unless otherwise specified.

The genomic characteristics of GBS0709 are summarized in Table 1. GBS0709 was almost identical to four *E. tarda* strains, with gANI values exceeding 99.1. Although *E. tarda* infection is a rare cause of bacterial gastroenteritis, wound infections, and bacteremia in humans (11, 12), the genome data of GBS0709 can help to increase our understanding of the virulence of *E. tarda*.

**TABLE 1:** Characteristics of the complete genome sequence of *E. tarda* strain GBS0709

Number of contigs	2
Structure	Circular chromosome and plasmid
Number of Miseq reads	2,556,215
Number of MinION reads [*N*_50_ value (bp)]	113,995 (13,864)
Total length (bp)	3,637,454
GC content (%)	57.3
Coverage (×)	260
*N*_50_ value (bp)	3,632,068
Number of coding sequences	3,310
Number of rRNAs	25
Number of tRNAs	87
Whole-genome sequence accession no.	AP028090 for the chromosome and AP028091 for the plasmid
DRA accession no.	DRX528540 for Illumina MiSeq data and DRX528541 for MinION data
gANI values of GBS0709 for *E. tarda* strains	
NBRC105688 (GCA_000341505.1)	99.3195
ATCC15947 (GCA_003113495.2)	99.2589
FDAARGOS_1473 (GCA_019933175.1)	99.2
ATCC15947 (GCA_000264805.1)	99.1671

## Data Availability

The complete genome sequence of *E. tarda* GBS709 is available at the National Center for Biotechnology Information under accession numbers AP028090 (chromosome) and AP028091 (plasmid). Raw sequence data from Illumina MiSeq and MinION are available at DDBJ Sequence Read Archive under DRA accession numbers DRX528540 and DRX528541.
